# Primary Breast Tuberculosis Mastitis Manifested as Nonhealing Abscess

**DOI:** 10.1055/s-0042-1749123

**Published:** 2022-06-14

**Authors:** Huu Hoang, Etienne El-Helou, Catalin-Florin Pop, Ammar Shall, Manar Zaiter, Jessica Naccour, Tran T. H. Nguyen, Xuan D. Ho, Van C. Nguyen

**Affiliations:** 1 Department of Oncology, Hue college of Medicine and Pharmacy—Hue University, Hue, Vietnam; 2 Department of Surgical Oncology, Institute Jules Bordet, Brussels, Belgium; 3 Department of Radiology, Institute Jules Bordet, Brussels, Belgium; 4 Department of Emergency Medicine, Hopital Erasme, Brussels, Belgium

**Keywords:** breast abscess, mycobacterium tuberculosis, extrapulmonary tuberculosis, breast tuberculosis, case report

## Abstract

Primary breast tuberculosis (TB) is a rare extrapulmonary TB mainly affecting young women of childbearing age from endemic countries. Its incidence is increasing in immunocompromised and HIV-infected people and with the emergence of drug-resistant strains of *Mycobacterium tuberculosis* (MTB). There are no specific clinical signs suggestive of this disease, it often presents as a hard mass or breast abscess. There is an overlap of features with other inflammatory, infectious, benign lesions, fat necrosis and malignant neoplasms of the breast. The detection of MTB remains the gold standard for diagnosis. Several other diagnostic modalities are used, with varying lack of sensitivity and specificity, and with a range of false negatives. A quarter of cases were treated solely on the basis of clinical, imaging or histological suspicion, without confirmation of the diagnosis. Therefore, we report the case of a young Vietnamese woman, presented for a nonhealing breast abscess, and diagnosed with breast TB based on the patient's ethnicity, histological findings, lack of clinical response to conventional antibiotic therapy, and a good clinical response to anti-TB treatment.

Tuberculosis (TB) is a worldwide health problem and is considered the second leading cause of death due to an infectious etiology . 1 *Mycobacterium tuberculosis* (MTB) bacillus is the pathogen, it survives in the host's macrophages 2 and can therefore affect any organ, 3 most frequently the lungs. 1

Extrapulmonary tuberculosis (EPTB) is difficult to diagnose and often based on clinical signs and medical history. Many patients begin anti-TB treatment without a definitive laboratory diagnosis. 4 The incidence of EPTB is 15% of TB worldwide. 4

The breast is an uncommon site of EPTB, it represents less than 0.1% of all breast pathologies. 1 2 It was first described in 1829 by Cooper. 3 They are very rare when considered primary, 2 that is, when we cannot demonstrate the presence of another TB site. 3 Due to the rarity of the disease and the overlap in features with breast carcinoma and pyogenic breast abscess, understanding the pathophysiology, assessment, and management is essential . 1

Here, we present a case of primary breast TB (BTB), manifested by nonhealing abscesses, diagnosed by exclusion based on the patient's country of origin and her response to treatment.

## Case Description

We present the case of a 32-year-old gravida twice, para twice, abortus 0 (G2P2A0) Vietnamese woman with no known food or drug allergy nor any significant medical/surgical history. She presented to the emergency department for swelling, induration, and mastodynia of the left breast. The pain started a day before the presentation. She denied any associated symptoms like fever, night sweats, weight loss or respiratory symptoms. There was no family history of breast cancer. She had no history suggestive of past or present TB or contact with a TB patient. She was not pregnant and had stopped breastfeeding 2 years ago.

On physical examination, the patient was febrile (temperature 38.7°C), tachycardic, normotensive, and had normal oxygen saturation (SpO _2_ 97%). Examination of the left breast revealed fixed breast induration with erythema associated with severe tenderness, but no associated secretions or palpable axillary lymph nodes. No nipple secretion or retraction reported.

**Fig. 1:**
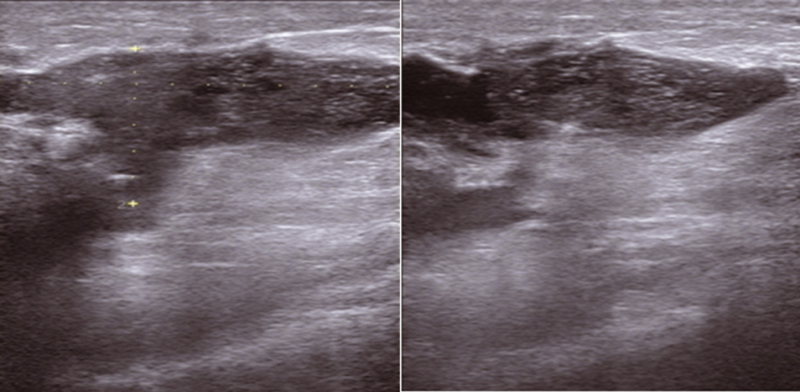
Large irregular heterogeneous hypoechoic collection measuring 20 mm in thickness in the upper quadrants of the left breast.

Laboratory tests revealed leukocytosis, increase in erythrocyte sedimentation rate, and elevated C-reactive protein (CRP). Ultrasound with antiradial view showed a large irregular heterogeneous hypoechoic collection, measuring 20-mm thickness in the upper quadrants of the left breast ( Fig. 1 ).

**Fig. 2:**
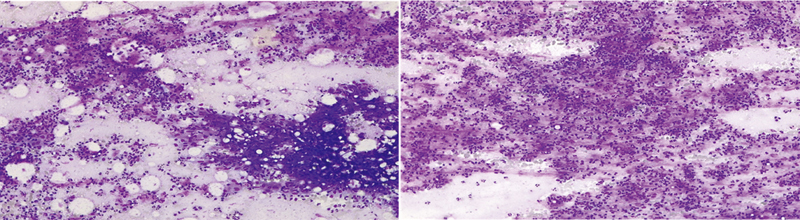
Granulomatous inflammation. Mixed inflammatory cell necrosis including lymphocytes, plasma cells, and macrophages.

Incisional drainage, debridement, and a biopsy were performed to rule out an underlying malignancy, and she was discharged on antibiotics. Cytobacterial culture was negative and pathology showed simple granulomatous inflammation ( Fig. 2 ). She was lost to follow-up after that.

**Fig. 3:**
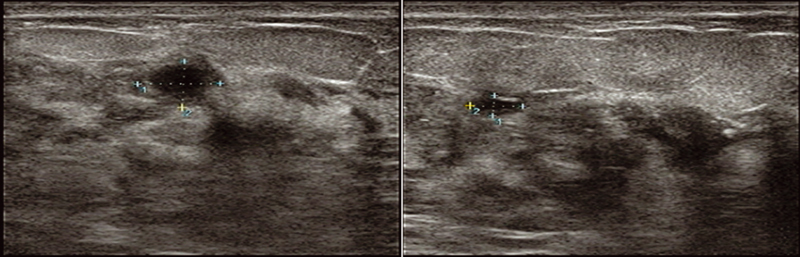
Ultrasound showing small hypoechoic collections.

Two months later, the patient presented with the same symptoms associated with purulent leak from old surgical scar site. Ultrasound showed four small hypoechoic collections in the left breast ranging in size between 10 and 30 mm ( Fig. 3 ).

A second time, the etiology was considered as a pyogenic abscess, thereafter the same attitude consisting of surgical debridement with antibiotic therapy was followed without improvement.

Given this presentation, the ethnic origin of the patient, histological results, lack of clinical response to conventional antibiotic therapy, and after reviewing the literature; TB polymerase chain reaction (PCR) on peripheral blood and Ziehl–Neelsen stain examination on the drained liquids were performed and were both negative.

However, we considered the rate of false-negatives and decided to initiate standard anti-TB treatment.

**Fig. 4:**
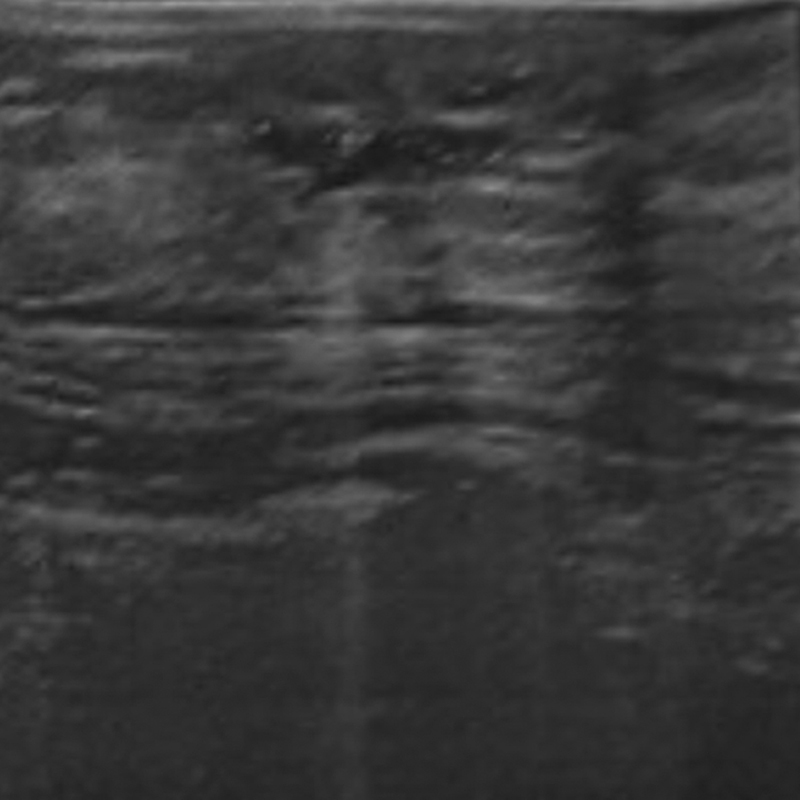
Small residual collection in the left breast.

Follow-up after 2 weeks showed good clinical improvement and a well-healed wound with no fluid discharge. Ultrasound showed only a small residual collection in the left breast ( Fig. 4 ). Normal chest X-ray and negative intradermic reaction ruled out associated pulmonary TB.

**Fig. 5:**
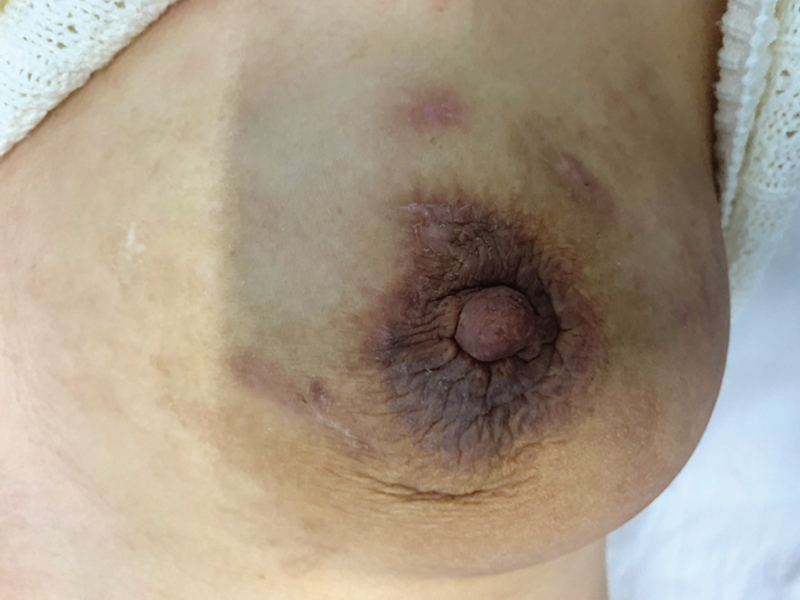
Breast photo after 2 months of anti-TB treatment. TB, tuberculosis.

On month 2 of treatment, the patient presented again for follow-up where we noted a total improvement in her condition. The skin has healed well with no signs of inflammation or infection, and the ultrasound showed no residual collection ( Fig. 5 ).

## Discussion

Breast tissue is highly resistant to mycobacteria. 1 2 It provides a barren atmosphere for the survival and proliferation of bacilli, like spleen and skeletal muscle, by means of the two immune modalities, innate by the macrophage which phagocytizes the bacilli, and cell-mediated T4-lymphocytes through cytokines they produce. 1

In West, the prevalence of BTB is less than 0.1% and it rises to 6.8% in other countries, 3 but its decreasing recently due to the progression of treatment and health care alongside improved socioeconomic status. 1 Due to its rarity in Western Europe, there is a lack of awareness which delays its diagnosis. This prompts the doctor to suspect breast TB in people from endemic countries, since 87% of cases concern migrant patients . 5

BTB is commonly found in women of reproductive age. 3 Increased vascularity of the breast with dilated ducts predisposes pregnant and lactating women to infection. 1 3 In addition the proinflammatory T-helper response is suppressed during pregnancy which may rise the predisposition to infection or reactivation of TB. 3 Multiparity is also considered a risk factor. 5 BTB incidence increases also in immunocompromised 1 2 and HIV infected people 3 and with the emergence of drug-resistant strains of MTB. 2

There are many ways in which the breast becomes infected with mycobacterial bacilli, whether through hematologic, lymphatic, or extensive spread from adjacent structures. 1 The primary form is the result of an infection that spreads through cracks in the nipple, abrasions of the skin of the breast, 2 or through the milk ducts. It can be transmitted directly through the infected palatal tonsils of breastfed infants. 1 Breast trauma is considered another risk factor. 2

There are no specific clinical signs suggestive of BTB. It commonly presents as a hard, nonspecific mass with irregular boundaries and attaching to surrounding tissue in 75% of cases. 2 3 Breast abscess comes in the second and it is present in 15% of cases. 3 It is mainly found in the central and upper outer quadrant of the breast. 2 The presentation may also be associated with nipple retraction, sinus, and fistula formation, and skin ulceration. 3 However, nipple discharge is rarely seen. Axillary lymphadenopathy is not always found, it can be seen in 50 to 75% of cases. 2 The patient may sometimes complain of mastalgia unrelated to the menstrual cycle. 3 Systemic symptoms are often not reported. 2 The average time to onset of symptoms varies between a few weeks and more than 7 months. 3 The presence of multiple lesions is not common, yet there are reported cases with bilateral lesions which represent up to 30% of cases. 2

The diagnosis of BTB remains difficult and sometimes requires rigorous examination and invasive procedures which leads to a delay in TB therapy 5 and administration of unnecessary and inappropriate treatment 3 because most often the diagnosis is wrong. 1 In addition, laboratory confirmation of the diagnosis in suspected cases is difficult due to limited resources and the low bacterial load of MTB bacilli, 4 the prevalence of which is between 0 and 38.6% 2 ; however, the culture of MTB and Ziehl–Neelsen stain remains the gold standard for diagnosis. 1 2 3 5 Several other diagnostic modalities are used which are often invasive with a varying lack of sensitivity and specificity and with a range of false negatives . 1

BTB comes in the form of three radiological images: diffuse, nodular, and sclerosing. The mammography may show skin retraction, trabecular thickening, and a poorly defined mass. Ultrasound is best used to assesses axillary lymphadenopathy and to guide percutaneous histological sampling and drainage. Computed tomography (CT) can be used to visualize the deep localization of the mass and evaluate thoracic wall, ribs, and bone involvement. Additionally, CT scan can visualize the opening of the fistula which can also be better visualized by magnetic resonance imaging (MRI). On MRI, the lesions of BTB are usually T2-hyperintense image. 2

Quaglio et al published a systemic review which demonstrated that about half of the cases had negative culture and PCR. 3 Also, McGuire et al performed a retrospective study of BTB summarizing the results of diagnosis used where 58% of Mycobacterial culture were negative, healthy tissue and nonspecific results were found in 27% of histological examinations, 26% of cytological examination were negative, a negative Ziehl–Neelsen stain was found in 94%, and the mycobacterial PCR was negative in all cases carried out. In addition, 25% of cases were treated based on clinical, imaging, or histological suspicion only, without confirmation of the diagnosis . 5

BTB is a difficult diagnosis to establish, as it overlaps a wide variety of differential diagnoses including inflammatory diseases, such as Wegener's granulomatosis and sarcoidosis; infectious, such as actinomycosis, brucellosis, and pyogenic abscesses; other benign lesions, such as fibroadenoma; fatty necrosis, as well as malignant neoplasms of the breast. 3

There are no specific guidelines for the treatment of BTB, all publications reported the application of standard TB treatment, with 2 months four-drug strategy of rifampicin, isoniazid, ethambutol, and pyrazinamide, followed by 6 to 7 months of two-drug rifampicin and isoniazid. Some cases that required prolongation up to 18 months, due to poor clinical response, are also reported. 3 Some reports have pointed to the emergence of resistance to this regimen; hence, adding second-line treatment of ethionamide, kanamycin, ofloxacin, and para-amino salicylic acid has shown a good response . 1

Drainage of abscesses prevents the formation of fistulas and sinuses. The residual fistula tract can be excised with a minimal normal tissue loss. 1 Mastectomy has been performed in 4% of cases, representing 4.5% of all breast surgeries in developing countries. 1

BTB has an excellent prognosis with a remission in more than 95% of cases and rare recurrences. 5

## Conclusion

BTB is a rare disease that mainly affects young women. Its diagnosis is often difficult despite the different modalities used. We urge all physicians to keep this differential diagnosis in mind, especially when faced with a patient presenting with a breast lump or abscess and having a history of migration from endemic countries. Treatment should not be delayed to avoid complications.

We encourage researchers to do studies that provide specific guidelines for the diagnostic treatment of BTB.

## References

[1] Sinha R Breast tuberculosis Indian J Tuberc 2019 66 01 6 11 30797285 10.1016/j.ijtb.2018.07.003

[2] Baykan A H Sayiner H S Inan I Aydin E Erturk S M Primary breast tuberculosis: imaging findings of a rare disease Insights Imaging 2021 12 01 19 33587199 10.1186/s13244-021-00961-3 PMC7884561

[3] Quaglio G Pizzol D Isaakidis P Breast tuberculosis in women: a systematic review Am J Trop Med Hyg 2019 101 01 12 21 31115305 10.4269/ajtmh.19-0061 PMC6609192

[4] Jørstad M D Dyrhol-Riise A M Aßmus J Marijani M Sviland L Mustafa T Evaluation of treatment response in extrapulmonary tuberculosis in a low-resource setting BMC Infect Dis 2019 19 01 426 31096926 10.1186/s12879-019-4034-z PMC6524265

[5] McGuire E Carey L Tiberi S Breast tuberculosis in East London: a 13-year retrospective observational study Breast J 2020 26 02 235 239 31486176 10.1111/tbj.13517

